# Glu-mGluR2/3-ERK Signaling Regulates Apoptosis of Hippocampal Neurons in Diabetic-Depression Model Rats

**DOI:** 10.1155/2019/3710363

**Published:** 2019-06-10

**Authors:** Zhuo Liu, Yuanshan Han, Hongqing Zhao, Weixu Luo, Ling Jia, Yuhong Wang

**Affiliations:** ^1^Hunan University of Chinese Medicine, Training Bases, Hunan Key Laboratory of Chinese Materia Medical Powder and Innovative Drugs Established by Province and Ministry, Changsha 410208, Hunan, China; ^2^First Hospital of Hunan University of Chinese Medicine, Changsha 410007, Hunan, China

## Abstract

**Objectives:**

Diabetes mellitus is frequently accompanied by depression (diabetes−depression, DD), and DD patients are at higher risk of diabetes-related disability and mortality than diabetes patients without depression. Hippocampal degeneration is a major pathological feature of DD. Here, we investigated the contribution of the Glu−mGluR2/3−ERK signaling pathway to apoptosis of hippocampal neurons in DD model rats.

**Methods:**

The DD model was established by high-fat diet (HFD) feeding and streptozotocin (STZ) injection followed by chronic unpredictable mild stress (CUMS). Other groups were subjected to HFD + STZ only (diabetes alone) or CUMS only (depression alone). Deficits in hippocampus-dependent memory were assessed in the Morris water maze (MWM), motor activity in the open field test (OFT), and depression-like behavior in the forced swim test (FST). Terminal deoxynucleotidyl transferase (TdT) dUTP nick-end labeling (TUNEL) was used to estimate the rate of hippocampal neuron apoptosis. Hippocampal glutamate (Glu) content was measured by high performance liquid chromatography. Hippocampal expression levels of mGluR2/3, ERK, and the apoptosis effector caspase-3 were estimated by immunohistochemistry and Western blotting.

**Results:**

DD model rats demonstrated more severe depression-like behavior in the FST, greater spatial learning and memory deficits in the MWM, and reduced horizontal and vertical activity in the OFT compared to control, depression alone, and diabetes alone groups. All of these abnormalities were reversed by treatment with the mGluR2/3 antagonist LY341495. The DD group also exhibited greater numbers of TUNEL-positive hippocampal neurons than all other groups, and this increased apoptosis rate was reversed by LY341495. In addition, hippocampal expression levels of caspase-3 and mGluR2/3 were significantly higher, ERK expression was lower, and Glu was elevated in the DD group. The mGluR2//3 antagonist significantly altered all these features of DD.

**Conclusions:**

Comorbid diabetes and depression are associated with enhanced hippocampal neuronal apoptosis and concomitantly greater hippocampal dysfunction. These pathogenic effects are regulated by the Glu−mGluR2/3−ERK signaling pathway.

## 1. Introduction

Among patients with diabetes mellitus, the prevalence of depression-like symptoms is 30%−50%, and the risk of suicide is 8 times higher than in the general population [1]. In addition, patients with comorbid diabetes−depression (DD) are more likely to experience disability-related work loss and mortality compared to patients with diabetes or depression alone [2, 3]. Therefore, uncovering the unique pathogenic mechanisms of DD is critical to improving patient outcome.

Diabetes is a group of metabolic diseases characterized by chronic blood glucose elevation due to insufficient insulin secretion or reduced insulin sensitivity [4]. Hyperglycemia in diabetes can also lead to increased plasma cortisol and glucagon. In turn, long-term elevation of cortisol can damage the hippocampus and promote the development of depression [5].

The hippocampus is the key structure necessary for associative learning and memory, and is highly prone to damage in a number of disease processes, including stress and depression [6, 7]. Hippocampal dysfunction is strongly associated with cognitive decline in diabetic patients [8]. Further, depression and anxiety may exacerbate insulin resistance [9], initiating a reciprocally reinforcing cycle of worsening metabolic disruption, depression, and cognitive deficits.

The strength of glutamatergic (Glu) neurotransmission is controlled by postsynaptic ionotropic Glu receptor trafficking, which in turn is strongly regulated by metabotropic glutamate receptor (mGluR) activity [10]. Recent studies have suggested that mGlu2/3 receptor blockers can act as antidepressants [3, 11, 12], thereby implicating mGluR2/3 in disease pathogenesis. Extracellular signal-regulated kinase (ERK) is activated under stressful and inflammatory conditions [13, 14] and modulates downstream pathways regulating apoptosis [14, 15]. Activated ERK is involved in a variety of disease processes, such as tumor formation [9], as well as in neuroprotection, neurogenesis, and differentiation [16]. There is also growing evidence that Glu transmission is involved in the pathogenesis of depression by activating ERK through metabotropic glutamate receptor 2/3 (mGluR2/3). Depressive behaviors can be induced by lowering the synthesis of neurotrophic factors, resulting in neuronal apoptosis [17]. However, it is unknown if mGluRs contribute to the hippocampal damage of DD through the ERK pathway. Therefore, this study examined activation of the Glu−mGluR2/3−ERK pathway and the relationships with apoptosis of hippocampus neurons and behavioral abnormalities in DD model rats. Considering the high incidence, mortality, and disability rate of DD, such results could define the Glu−mGluR2/3−ERK pathway as an important therapeutic target.

## 2. Materials and Methods

### 2.1. Animal Materials

Male Sprague Dawley (SD) rats [SCXK (xiang) 2013-0004] weighing 180−200 g were obtained from Hunan SJA Laboratory Animal Co. Ltd. Rats were housed in the specific pathogen-free (SPF) Laboratory Animal Center of Hunan University of Chinese Medicine at a controlled temperature (25 ± 2°C) and humidity (50% ± 5%) under a 12 h/12 h light/dark cycle for five days before experiments. All animal experiments were conducted in accordance with the requirements of the Guidelines for Laboratory Animals and Their Use (NIH No. 8023 Publication, Revised in 1996) of the National Institute of Health and approved by the Animal Ethics Welfare Committee of the First Affiliated Hospital of Hunan University of Traditional Chinese Medicine.

### 2.2. Animal Grouping and Modeling

Eighty-five SD rats were first divided into three groups, a diabetes model group (n=55, set to ensure sufficient numbers for subsequent treatments described below), normal group (n = 20), and depression-like model group (n = 10). The diabetes model group was fed a high-fat diet (10% cholesterol 10 mL/kg) by gavage for 14 days. After overnight fasting, animals were injected intraperitoneally with 38 mg/kg streptozotocin (STZ) freshly dissolved in 0.1 mol/L citrate buffer (Sigma-Aldrich Co., USA). The normal group and depression-like model group were injected with 2 mL/kg of 0.1 mol/L citrate buffer as a control.

Fasting blood glucose was tested 72 h after STZ injection. Rats that survived the injection with blood glucose over 16.70 mmol/L were selected for subsequent treatments to establish a diabetes alone group (n = 15), DD group (n = 25), and LY341495+DD group (n = 15).

The depression-like model group, DD model group, and LY341495+DD group were exposed to 28 days of chronic unpredictable mild stress (CUMS) according to the methods of Willner (year) with modifications. The stress conditions included a 4°C ice water bath for 5 min, 45°C hot water bath for 5 min, 50 V electric shock, clip tail for 1 min, reversal of the day−night cycle for 24 h, and wet cage housing for 24 h. Stress conditions were presented daily in random order. The depression-like model group and DD group were injected daily with normal saline, while the LY341495+DD group was injected daily with 1 mg/kg LY341495 in normal saline starting on the first day of CUMS. Body weight and blood glucose were assessed weekly starting from the seventeenth day of CUMS.

After modeling, rats were examined in the Morris water maze (MWM), open field test (OFT), and forced swim test (FWT) as described below. Following testing, rats were fasted for 24 h, anesthetized with 4 mL/kg 10% chloral hydrate, and sacrificed for isolation of the hippocampi. One hippocampus was frozen in liquid nitrogen for Glu analysis and the other fixed in paraformaldehyde for histochemistry.

### 2.3. Reagents and Equipment

Glutamate control products were obtained from China Pharmaceutical Biological Products Testing Institute, LY341495 from Sigma (St. Louis, MO, USA), monoclonal antibodies against mGluR2/3, ERK, and GFAP from Abcam (Cambridge UK), and secondary antibody kits, a DAB kit, and imported sheep serum working solution from Beijing Zhongshanjinqiao (China). The MWM video tracking system was from Panlab (Barcelona, Spain), inverted microscope from Olympus (Tokyo, Japan), high performance liquid chromatography system from Agilent (Santa Clara, CA, USA), tissue slicer from Themo (MA, USA), protein weight marker set (10−170 kD) from THERMO SCIENTIFIC (MA, USA), and anti-GAPDH as a gel loading control from ProteinTech (Colo, USA).

### 2.4. Behavioral Tests

#### 2.4.1. Forced Swimming Test (FST)

The FST was used to assess depression-like behavior [18]. The forced swimming apparatus was a cylindrical plastic bucket with a diameter of 20 cm and a height of 50 cm filled with water to a depth of about 40 cm to ensure that the rats could not touch the bottom. The water temperature was kept at about 24°C. Each rat was placed in the bucket for 6 minutes, and the times spent swimming and passively floating were recorded by video camera. Passive floating time is an index of depression-like behavior.

#### 2.4.2. Morris Water Maze Test

The MWM was used to assess hippocampus-dependent spatial learning and memory [19]. Experiments began with trainings trials on day 17. Rats were placed in the pool facing the water and allowed to search for platform (2 cm below the water level). If the platform was not found within 60 s, the rat was guided to the platform and allowed to stay for 10 s. On day 22, probe trials were conducted in which the platform was removed and the time spent in the platform (target) quadrant measured over 60 s as a test of spatial memory.

#### 2.4.3. Open Field Test

The rats were placed in the same corner of a black open box divided into 25 smaller areas [20]. After a 1-min adaptation period, horizontal movements as indicated by transition of all four feet into a new area and the number of rearings were counted over 4 min.

### 2.5. TUNEL Staining

Hippocampal tissue was fixed, paraffin embedded, sectioned (), heated at 60°C for 10 min, deparaffinized in xylene for 5 min, rehydrated in graded ethanol (100%, 95%, 80%, 75%) and DDW (1 min per solution), permeabilized in PBS with 0.1% Triton X–100 for 8 min, and washed twice in PBS (10 min/wash). Sections were then incubated in TUNEL reaction solution under darkness in a wet box for 60 min at 37°C, stained with DAB, counterstained with hematoxylin, and mounted on slides with antifade medium. Five fields were randomly selected for quantitation of TUNEL-positive hippocampal neurons.

### 2.6. Glutamate Content

The frozen hippocampus was weighed and homogenized in methanol. The lysate was centrifuged and the supernatant recovered for HPLC analysis of Glu using an amino acid analysis column (250 mm × 4 mm, 5 *μ*m) and the following conditions: mobile phase containing 5% sodium methanol acetate:acetonitrile (80:20), flow rate of 0.2 ml/min, sample volume 20 *μ*L, and column temperature of 40°C. Peaks were quantified against a standard curve constructed using Glu solutions of 3.13, 0.781, 0.195, 0.0488, and 0.0122 mg/mL. According to the peak area, the standard curve equation was y = 75.09x + 34.63 and the correlation coefficient was 0.9978.

### 2.7. Immunohistochemistry

Tissues were embedded in paraffin, sectioned, dehydrated in an ethanol gradient (75%, 95%, 100%), deparaffinized in xylene, rehydrated, washed in PBS, blocked in goat serum for 30 min at 37°C, and incubated overnight at 4°C with antibodies against caspase-3 (1:50), mGluR2/3 (1:100), and (or) ERK (1:100). Immunostained sections were then incubated in secondary antibody kit reaction enhancement solution for 10 min and secondary antibody for 10 min, followed by rising and DAB staining. Finally, sections were rehydrated, hyalinized, mounted, and digitally photographed under high magnification (×400). Integral optical density (IOD) as a measure of average immunopositivity was analyzed using Image Pro software.

### 2.8. Western Blotting of Hippocampal mGluR2/3, ERK, and Caspase-3

The hippocampi were removed, homogenized, lysed in extraction buffer, flash-frozen, and stored until analysis. After thawing, total protein content was determined by bicinchoninic acid (BCA) assay. Proteins were separated on 12% polyacrylamide gels and transferred to PVDF membranes. Membranes were blocked in skimmed milk and probed with antibodies against mGluR2/3 (1:1000), ERK (1:2000), caspase-3 (1:500), and GAPDH (1:5000) as the gel loading control. Target protein expression levels were quantified using ImageJ.

### 2.9. Statistical Analysis

All data are expressed as mean ± standard deviation (SD). Statistical testing was conducted using SPSS 19. Differences among group means were evaluated by one-way ANOVA followed by Tukey's multiple comparison tests for pairwise comparisons. Paired group means were compared by independent samples t test. A p < 0.05 (two-tailed) was considered statistically significant for all tests.

## 3. Results

**Figure 1:**
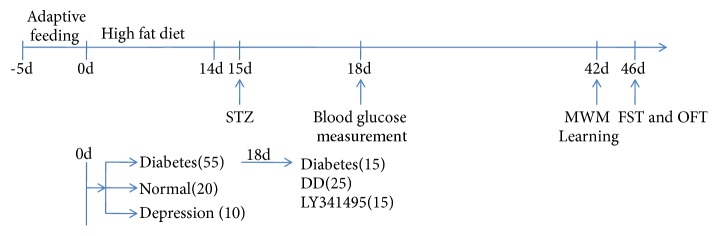
Timeline of the experimental procedures. DD: diabetes plus depression, MWM: Morris water maze, STZ: streptozotocin.

Of the 85 rats used in this study, 6 were removed because of failure to induce diabetes (n = 2) or DD (n = 4). The experimental protocols are summarized schematically in Figure 1.

### 3.1. Augmented Spatial Memory Deficits and Depression-Like Behavior in DD Rats

**Figure 2:**
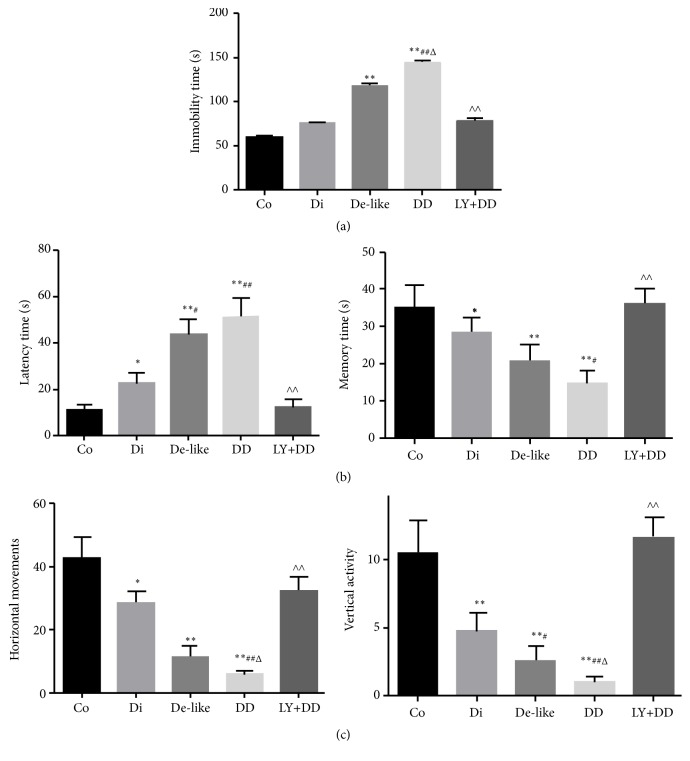
Exacerbation of behavioral deficits in DD rats and amelioration by mGluR blockade. All results are presented as mean ± standard deviation (n=10 rats/group). Co: control group; Di: diabetes model group; De-like: depression-like model group; DD: DD model group; LY+DD: DD model treated with LY341495. (a) Depression-like behavior (immobility) in the forced swimming test. (b) Spatial learning deficits (left panel) and memory deficits (right panel) as assessed by the Morris water maze. (c) Reduced horizontal and vertical activity in the open field test. ^*∗*^*p* < 0.05, ^*∗∗*^*p* < 0.01 vs. control; ^#^*p* < 0.05, ^##^*p* < 0.01 vs. diabetes group; ^Δ^*p* < 0.05, ^ΔΔ^* p* < 0.01 vs. depression group, ^∧∧^*p* < 0.01 vs. DD group.

In the FST, immobility time was significantly higher in the depression-like alone and the DD groups than the normal control group and diabetes alone groups, indicating successful induction of a depression-like phenotype (Figure 2(a)). This enhanced depression-like behavior was reversed to near control levels in the DD group by treatment with the mGluR2/3 antagonist LY341495 during CUMS. The DD group also took significantly longer to find the platform in MWM training trials (Figure 2(b), left panel) and spent significantly less time (% of 60 s) in the target quadrant (Figure 2(b), right panel) during probe trials compared to the normal control, depression-like alone, and diabetes alone groups, indicating greater disruption of spatial learning and memory. Like the behavioral signs of depression-like, deficits in spatial learning and memory were reversed by LY341495. The numbers of horizontal movements and rearings were significantly reduced in the DD group compared to the control, depression-like alone, and diabetes alone groups, and again these deficits were significantly reversed by LY341495 (Figure 2(c)).

### 3.2. Facilitation of Hippocampal Neuron Apoptosis in DD Rats

**Figure 3:**
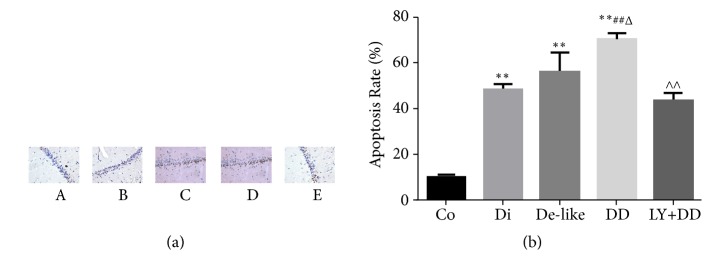
TUNEL staining showing enhanced apoptosis of neurons in the hippocampus of DD rats. (a) Images of TUNEL staining (×400). Co: control group; Di: diabetes model group; De-like: depression-like model group; DD: DD model group; LY+DD: LY341495+DD group. (b) Quantification of TUNEL-positive cell number. Results are presented as mean ± standard deviation (n=6 rats/group). ^*∗*^*p* < 0.05, ^*∗∗*^*p* < 0.01 vs. control; ^#^*p* < 0.05, ^##^*p* < 0.01 vs. diabetes group; ^Δ^*p* < 0.05, ^ΔΔ^*p* < 0.01 vs. depression group; ^∧^*p* < 0.05, ^∧∧^*p* < 0.01 vs. DD group.

In hippocampal sections from control animals, neurons were neatly arranged with clear shapes, and TUNEL staining was lower (Figure 3). All three model groups demonstrated significantly higher rates of TUNEL staining, with the highest rate in the DD group. These degenerative changes in the DD group were largely reversed by LY341495 treatment during CUMS.

### 3.3. Enhanced Glu Content in the Hippocampus of DD Rats

**Figure 4:**
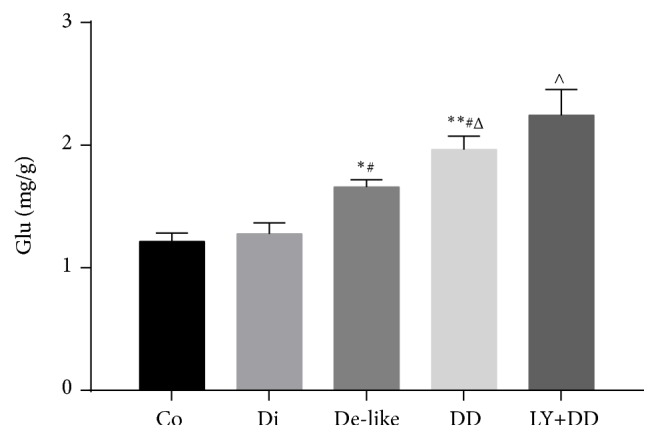
Changes in hippocampal glutamate (Glu) content among groups. All results are presented as mean ± standard deviation (n=6 rats/group). Co: control group; Di: diabetes model group; De-like: depression-like model group; DD: DD model group; LY+DD: LY341495+DD group. ^*∗*^*p* < 0.05, ^*∗∗*^*p* < 0.01 vs. control; ^#^*p* < 0.05, ^##^*p* < 0.01 vs. diabetes group; ^Δ^*p* < 0.05, ^ΔΔ^*p* < 0.01 vs. depression group; ^∧^*p* < 0.05, ^∧∧^*p* < 0.01 vs. DD group.

HPLC results revealed significantly elevated Glu content in hippocampi of the depression-like alone and DD groups (Figure 4) compared to the normal control group and even higher content in the LY341495+DD group.

### 3.4. Enhanced Expression of Caspase-3 in the Hippocampus of DD Group Rats

**Figure 5:**
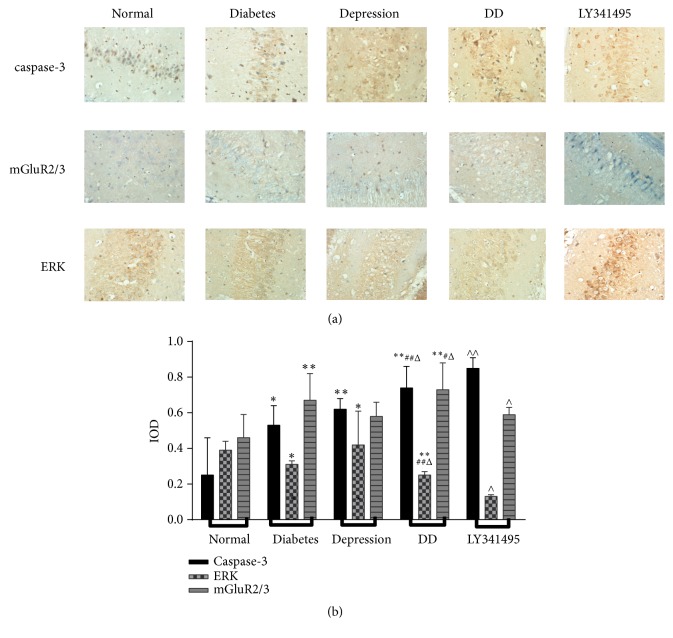
Changes in immunoexpression of caspase-3, mGluR2/3, and ERK among model groups. (a) Images of immunohistochemical staining (×400). (b) Quantification of immunoexpression by integral optical density (IOD). Results are presented as mean ± standard deviation (n=6 rats/group). A: control group; B: diabetes model group; C: depression-like model group; D: DD model group; E: LY341495+DD. ^*∗*^*p* < 0.05, ^*∗∗*^*p* < 0.01 vs. Control; ^#^*p* < 0.05, ^##^*p* < 0.01 vs. diabetes group; ^Δ^*p* < 0.05, ^ΔΔ^*p* < 0.01 vs. depression group; ^∧^*p* < 0.05, ^∧∧^*p* < 0.01 vs. DD group.

**Figure 6:**
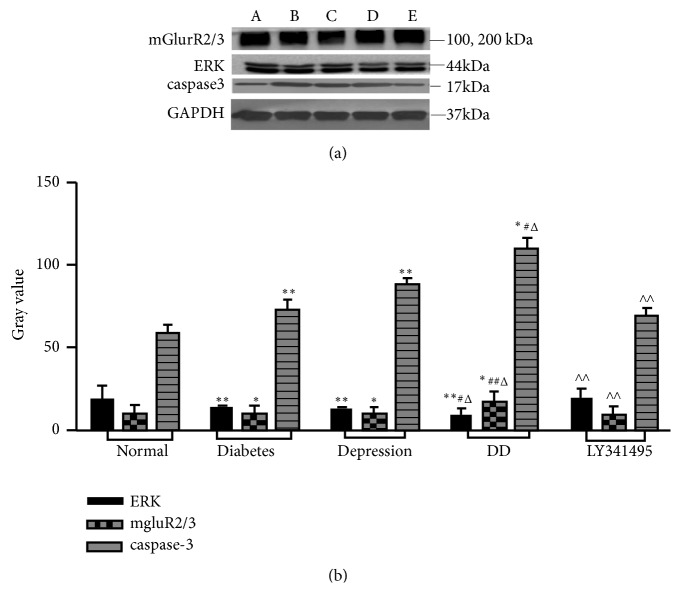
Changes in hippocampal expression of caspase-3, mGluR2/3, and ERK among model groups according to Western blot analysis. (a) Images of Western blots. (b) Densitometric quantitation. Results are presented as mean ± standard deviation (n=6 rats/group). A: control group; B: diabetes model group; C: depression-like model group; D: DD model group; E: LY341495+DD. ^*∗*^*p* < 0.05, ^*∗∗*^*p* < 0.01 vs. control; ^#^*p* < 0.05, ^##^*p* < 0.01 vs. diabetes group; ^Δ^*p* < 0.05, ^ΔΔ^* p* < 0.01 vs. depression group; ^∧^*p* < 0.05, ^∧∧^*p* < 0.01 vs. DD group.

Caspase-3 is the key effector enzyme for apoptosis. Caspase-3 immunoreactivity in the hippocampus was significantly enhanced in depression-like, diabetes, and DD groups (Figure 5). Western blotting confirmed significantly elevated caspase-3 expression in the hippocampus of all model groups and also showed partial reversal by LY341495 treatment (Figure 6).

### 3.5. mGluR2/3–ERK Regulates Apoptosis of Hippocampus Neurons in Model Rats

Metabotropic glutamate receptor 2/3 regulates neuronal activity by influencing ionotropic receptor trafficking. All model groups demonstrated enhanced expression of mGluR2/3 in the hippocampus compared to normal controls as revealed by immunohistochemistry and Western blotting. Conversely, the DD showed reduced ERK activity, which was increased by LY341495, suggesting that mGluR2/3−ERK regulates the apoptosis of hippocampus neurons.

## 4. Discussion

The global incidence of diabetes mellitus is rising, resulting in parallel increases in associated comorbidities such as cardiovascular disease, neuropathy, and depression. Many studies have confirmed that the prevalence of depression is higher in diabetic patients than healthy matched controls [21]. Indeed, up to 60%−75% of diabetes mellitus patients show signs of depressive behavior and 10%−35% have clinical depression [22, 23]. The mortality rate of diabetes mellitus is significantly higher when accompanied by depression [24], so it is crucial to elucidate the mechanisms underlying this enhanced susceptibility. Diabetic microangiopathy can lead to degeneration of hippocampal structure and function, as well as hypothalamic−pituitary−adrenal (HPA) axis dysfunction [25]. Chronic stress is also associated with hyperactivation of the HPA axis [6], and the resulting cortisol elevation can inhibit neurogenesis and damage hippocampal neurons. In turn, neuronal loss or dysfunction within the hippocampus can lead to depression [26]. Thus, diabetes may enhance the susceptibility to depression through neurovascular dysfunction and HPA hyperactivity, which can both damage the hippocampus directly and enhance the propensity from stress-induced damage.

Spontaneous activity in the OFT and spatial memory in the MWM were substantially impaired, while depression-like behavior in the FST (immobility time) was significantly increased in DD mice. These behavioral signs were accompanied by greater TUNEL staining, a sign of apoptosis induction [27] and disrupted cytoarchitecture in the hippocampus of DD rats. In accord with greater apoptosis rate among hippocampal neurons in DD rats, expression of caspase-3, the key effector protease for apoptosis [28], was enhanced as evidenced by both immunoexpression and Western blotting. In addition to TUNEL positivity, the hippocampus of DD mice exhibited absent neurons, cell swelling, nuclear pyknosis, vacuolar degeneration, and other pathological signs. It is suggested that these pathological changes in DD rats are related to apoptosis.

In diabetes, hippocampal Glu content is increased and mGluR2/3 subtype receptors are hyperstimulated [29]. Stimulation of mGluR2/3 activates the downstream ERK signaling pathway. Phosphoactivated ERK enters the nucleus, where it regulates genes involved in the cell cycle and apoptosis [30] and the synthesis and secretion of neurotrophic factors [31]. In this study, hippocampal Glu and mGluR were significantly elevated in DD rats compared to controls, while expression of ERK was reduced. Furthermore, hippocampal apoptosis, memory deficits, and depressive behavior in DD rats were reversed by LY341495, a specific antagonist of mGluR2/3 [32]. Administration of LY341495 to DD rats further enhanced Glu content in the hippocampus and reversed both the decrease in ERK and the increase in mGluR2/3. Previous studies have found antidepressant effects of mGluR2/3 antagonists as evidenced by reduced immobility time of mice in the FST [33, 34]. Moreover, mGluR2/3 increases extracellular dopamine in the nucleus accumbens and dose-dependently enhances cortical BDNF [10, 35], both of which are believed to be cardinal therapeutic effects of other antidepressant therapies. Further, these antidepressant actions were achieved in the present study by intraperitoneal injection of 1 mg/kg, which had been shown to have optimal antagonist efficacy [36, 37].

ERK expression was significantly reduced in the DD model group, while mGluR2/2 expression and Glu content were increased. This suggests that ERK expression is suppressed by mGluR2/3 hyperactivity in the hippocampus of DD rats. In turn, reduced ERK activity could disinhibit regulators of apoptosis and suppress neurotrophic factor expression, resulting in apoptosis and neurodegeneration, ultimately leading to depression-like behavior. This study provides evidence that dysregulation of the Glu−mGlur2/3−ERK pathway is directly involved in the pathogenesis of depression in diabetes and thus is a potentially valuable target for therapeutic intervention.

## Data Availability

The data used to support the findings of this study are available from the corresponding author upon request.
